# Toxicity Reduction after Craniospinal Irradiation via Helical Tomotherapy in Patients with Medulloblastoma: A Unicentric Retrospective Analysis

**DOI:** 10.3390/cancers13030501

**Published:** 2021-01-28

**Authors:** Anil Öztunali, Khaled Elsayad, Sergiu Scobioala, Mohammed Channaoui, Uwe Haverkamp, Oliver Grauer, Ronald Sträter, Angela Brentrup, Walter Stummer, Kornelius Kerl, Hans Theodor Eich

**Affiliations:** 1 Radiation Oncology Department, University Hospital Muenster, 48149 Muenster, Germany; anil.oeztunali@gmail.com (A.Ö.); Sergiu.Scobioala@ukmuenster.de (S.S.); Mohammed.Channaoui@ukmuenster.de (M.C.); Uwe.Haverkamp@ukmuenster.de (U.H.); hans.eich@ukmuenster.de (H.T.E.); 2 Neuro-Oncology Department, University Hospital Muenster, 48149 Muenster, Germany; oliver.grauer@ukmuenster.de; 3 Pediatric Oncology Department, University Hospital Muenster, 48149 Muenster, Germany; ronald.straeter@ukmuenster.de (R.S.); Kornelius.Kerl@ukmuenster.de (K.K.); 4 Department of Neurosurgery, University Hospital Muenster, 48149 Muenster, Germany; Angela.Brentrup@ukmuenster.de (A.B.); Walter.Stummer@ukmuenster.de (W.S.)

**Keywords:** conventional, Tomotherapy, intensity-modulated, toxicity

## Abstract

**Simple Summary:**

Medulloblastoma (MB) is one of the most common pediatric brain tumors. Surgical resection, followed by radiotherapy (RT) and chemotherapy, are the standard of care for medulloblastoma patients. This retrospective analysis assesses the toxicity profile of different radiation techniques (Tomotherapy versus conventional radiotherapy technique) and estimates survival rates. Radiotherapy via Tomotherapy seems to be an efficacious treatment for patients with lower rates of acute upper gastrointestinal and central nervous system toxicities than conventional radiotherapy techniques.

**Abstract:**

*Objectives*: Recent trials with craniospinal irradiation (CSI) via helical Tomotherapy (HT) demonstrated encouraging medulloblastoma results. In this study, we assess the toxicity profile of different radiation techniques and estimate survival rates. *Materials and Methods*: We reviewed the records of 46 patients who underwent irradiation for medulloblastoma between 1999 and 2019 (27 conventional radiotherapy technique (CRT) and 19 HT). Patient, tumor, and treatment characteristics, as well as treatment outcomes—local control rate (LCR), event-free survival (EFS), and overall survival (OS)—were reviewed. Acute and late adverse events (AEs) were evaluated according to the Radiation Therapy Oncology Group and the European Organization for Research and Treatment of Cancer (RTOG/EORTC) criteria. *Results*: In total, 43 courses of CSI and three local RT were administered to the 46 patients: 30 were male, the median age was 7 years (range 1–56). A median total RT dose of 55 Gy (range 44–68) and a median CSI dose of 35 Gy (range, 23.4–40) was delivered. During follow-up (median, 99 months), six patients (13%) developed recurrence. The EFS rate after 5 years was 84%. The overall OS rates after 5 and 10 years were 95% and 88%, respectively. There were no treatment-related deaths. Following HT, a trend towards lower grade 2/3 acute upper gastrointestinal (*p* = 0.07) and subacute CNS (*p* = 0.05) toxicity rates was detected compared to CRT-group. The risk of late CNS toxicities, mainly grade 2/3, was significantly lower following HT technique (*p* = 0.003). *Conclusion*: CSI via HT is an efficacious treatment modality in medulloblastoma patients. In all, we detected a reduced rate of several acute, subacute, and chronic toxicities following HT compared to CRT.

## 1. Introduction

Medulloblastoma (MB) is a malignant, embryonal solid tumor in the cerebellum. This neuroectodermal cancer is one of the most common pediatric brain tumors as it represents 10–20% of all central nervous system (CNS) neoplasms in children [1,2]. However, it can occur at any age, with an incidence peak between 3 and 7 years of age [3]. 

The multimodal treatment strategy (surgical resection of the primary tumor, followed by radiotherapy (RT) and chemotherapy) is the standard of care for medulloblastoma. According to risk groups, the 5-year survival rates range from 30% up to 90% [4,5,6,7,8,9,10,11]. These high survival rates highlight the importance of long-term treatment-related sequelae that significantly impact the patient’s quality of life. Thus, it is crucial to minimize any undesirable toxicities of medulloblastoma therapy [12].

Owing to the risk of metastasis at diagnosis (approximately 30%), medulloblastoma patients require craniospinal irradiation (CSI) [3,13,14]. A local radiotherapy boost to the posterior cranial fossa and dose escalation to residual tumor areas or metastases is mandatory [15,16,17]. CSI is a challenging indication as it is challenging to achieve a homogenous RT dose distribution with adequate target volume coverage while sparing organs at risk [18,19]. The conventional treatment technique is realized by a linear accelerator, which uses three-dimensional conformal radiotherapy (3DCRT) [20,21] and was later complemented by intensity-modulated radiotherapy (IMRT) [22,23,24]. With the 3DCRT-technique, typically, the neuroaxis is irradiated by using two opposing lateral fields on the cranium and up to two posterior spinal fields, depending on the height of the patient [20,21,25,26]. A new treatment strategy emerged with the introduction of the helical Tomotherapy (Accuray Inc, Sunnyvale, CA, USA) where large target volumes can be irradiated continuously and homogenously without field matching [13,18,20,27,28,29]. Additionally, HT is executed in supine position, which is favorable for nontarget tissue sparing, patient comfort, and dose conformity [30,31,32] with a homogenous dose distribution compared to 3DCRT [13,18,21,27,29,33]. 

As the treatment modalities of medulloblastoma are sophisticated, and the survival rates are higher than ever before, minimizing radiation-related sequelae now significantly impacts radiation planning and delivery. This analysis aims to assess HT’s clinical benefits compared to 3DCRT and compare the survival rates of both groups.

## 2. Materials and Methods

### 2.1. Selection Criteria

We conducted a retrospective analysis of patients diagnosed with medulloblastoma and treated with CSI at the department of radiotherapy and radiation oncology of the university hospital of Münster between the years 1999 and 2019. Forty-six patients with a primary diagnosis of medulloblastoma, treatment of the disease with CSI, and ≥6 months follow-up were included. Thirty (65%) males and 16 (35%) females were registered. The median age of the patients enrolled at diagnosis was 7 years (range 1–56). All patients treated after 2011 were irradiated via Tomotherapy. We grouped our patients according to the applied irradiation technique. There were 19 (41%) in the HT-group where patients underwent CSI with helical Tomotherapy and 27 (59%) in the CRT-group with patients treated conventionally between 1999 and 2011, with the linear accelerator Primus (Siemens, Erlangen, Germany).

### 2.2. Treatment Planning

Planning CT images from the vertex to the coccyx were acquired using the Aquilion LB CT-scanner (Canon Medical Systems, Otahara, Japan) with a slice thickness of 3 mm. Patients were immobilized in a supine position with thermoplastic masks for head support and vacuum cradles for body support and anesthetized if needed. 

All CT images were transferred via DICOM-RT protocol to the TomoTherapy treatment planning station in the HT-group and the Eclipse (Varian, Palo Alto, CA, USA) treatment planning station in the CRT-group for delineation of organs at risk (OAR) and planning target volumes (PTV). A radiation oncologist contoured OAR and PTVs. OAR included the brain, eyes, lenses, optical nerves, optic chiasm, thyroid, pituitary gland, heart, lungs, liver, kidneys, testis or ovaries, uterus, and breasts. 

### 2.3. Radiotherapy

In the CRT-group, treatment plans were delivered by the Primus linear accelerator using 6 MV photons. Two lateral opposed whole brain fields and, depending on the patient’s height, one to two posterior spinal fields were therefore planned. The two lateral cranial fields’ junction with the posterior spinal field was matched via collimator rotation and couch rotation to avoid cold spots and hot spots and enhance dose homogeneity. In cases with two spinal fields, the field junction was matched via fix gap calculation. Additionally, the feathering technique was applied with field junction match line shifts every five fractions. The distance of one shift was according to the length of one vertebra of the patient. Patients were immobilized in the prone position.

In the HT-group, all treatment plans were delivered by a Helical TomoTherapy HiArt II system treatment unit while all patients were immobilized in the supine position. Daily megavoltage computed tomography (MVCT) was used to confirm patient positioning prior to irradiation. The 6 MV photons were used here as well to irradiate the PTV. The plans were generated so that at least 95% of the PTV would receive the prescribed dose while surrounding organs at risk would receive a minimum dose below their tolerance limits. 

### 2.4. Toxicities

We collected data in the patients’ documents for acute and late toxicities based on the RTOG/EORTC criteria according to a department-specific register.

We collected baseline data for acute toxicities before initiation of radiotherapy, maximum toxicities during radiotherapy, and maximum toxicities up to 30 days after completion of radiotherapy, then every 3 months in the first 2 years. For late toxicities, we collected data years after the completion of radiotherapy. 

### 2.5. Statistical Analysis

Time-dependent event curves were generated by the Kaplan–Meier method and compared with log-rank tests. Overall survival rates (OS) were calculated from the first day of radiotherapy to the patient’s death date from any cause. Event-free survival rates (EFS) were calculated from radiotherapy initiation to the time of documented recurrent or progressive disease or death. Differences were considered statistically significant at a *p*-value < 0.05. Independent variables were first analyzed with univariate analysis. Variables shown by univariate analysis to be associated with EFS or OS were entered into a Cox proportional hazards regression model for multivariate analysis. Chi-squared or Fisher exact tests were performed to probe the relationship between pairs of categorical variables. The two-sample U-test was used to study the relationship between a categorical variable and a continuous variable. All statistical analyses were conducted with IBM SPSS Statistics 27.0 software (SPSS Inc., Chicago, IL, USA). 

## 3. Results

Thirty males (65%) and 16 females (35%) were analyzed (Table 1). Most of the patients were treated according to the German HIT trials for medulloblastoma (HIT2000 trial n = 31, 67%). Concurrent vincristine was applied to 39 patients with no difference between CRT and HT groups (85% vs. 84%, *p* = 1). All patients received maintenance treatment after irradiation. The CSI’s median radiation dose was 35 Gy (range, 23.4–40) and was 55 Gy (range, 44–68) for the posterior fossa or tumor bed. The median treatment time was 53 days (range, 28–62), and the median follow-up for the whole cohort was 99 months (range, 5–249). In patients receiving conventional RT there was a more prolonged follow-up compared to patients treated via helical Tomotherapy (116 months vs. 29 months, *p* < 0.001). Seven patients (15%) had metastasis at diagnosis. In the conventional RT group, four patients had metastatic medulloblastoma (2 M1, 1 M2, and 1 M3). In the HT-group, three patients had a metastatic lesion in the brain as well (M1).

**Figure 1:**
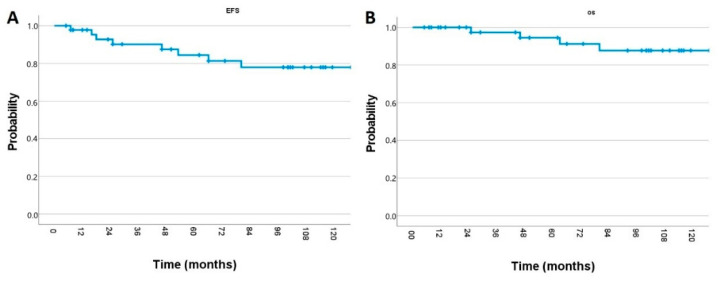
Kaplan–Meier estimate of (**A**) event-free survival and (**B**) overall survival of medulloblastoma patients.

After 2, 5, and 10 years, the overall EFS rates were 93%, 84%, and 78%, respectively (Figure 1A). We detected a significant difference in EFS rates between the HIT2000 protocol and other regimens (93% vs. 60%, *p* = 0.001). Regarding the radiation technique, the 5-year EFS-rate in the CRT-group was higher compared to the HT-group (93% vs. 67%). However, in the subgroup analysis for patients treated using HIT2000 protocol, there was no significant difference between the CRT-group (n = 24) and the HT-group (n = 7) (92% vs. 100%, *p* = 0.5). Regarding initial metastasis, there was no significant impact on EFS (85% vs. 80%, *p* = 0.9), and there was no impact of total gross resection on the 5-year EFS (86% for M0 vs. 84% for M+, *p* = 0.8). Female patients had a trend for favorable 5-year EFS rates compared to males (100% vs. 76%, *p* = 0.1).

For all patients, median OS was not reached. The overall OS rates after 2, 5, and 10 years were 100%, 95%, and 88%, respectively (Figure 1B). According to the treatment protocol, there was no significant difference in OS (97% with HIT2000 protocol and vs. 86% with other regimens, *p* = 0.1). The 5-year OS in the CRT-group was 96% and 88% in the HT-group (*p* = 0.6). There was no significant impact on initial metastasis on 5-year OS (97% vs. 80%, *p* = 0.6). Similarly, total gross resection (95% vs. 94%, *p* = 0.7) and gender (100% vs. 91%, *p* = 0.5) did not impact the 5-year OS rates. During follow-up examinations, six patients (13%) developed a recurrence. There were no significant differences between the different radiation techniques (three relapses in the HT-group vs. three relapses in the CRT-group, *p* = 0.7). The median TTP was 22 months (range 7–66). For patients treated with the conventional technique, the TTP was 53 (range, 25–66) months and 16 (range, 7–18) months for the HT-group. At the last follow-up, four patients were dead (two of them died after medulloblastoma relapse and two of them died due to myelodysplastic syndrome and glioblastoma), and four patients were alive with relapse. 

### Toxicities

During initial CSI courses, all patients experienced mild toxicities. The most common adverse effects (AEs) were fatigue, skin reactions, and CNS toxicity (i.e., headache, nausea, vomiting). The most common long-term toxicities were skin reactions (hyperpigmentation and alopecia), and CNS/sensorineural hearing loss. There were no treatment-related deaths. No unintended treatment break of ≥4 weeks occurred during RT course. All acute, subacute, and late radiation reactions are listed in Table 2. 

**Figure 2:**
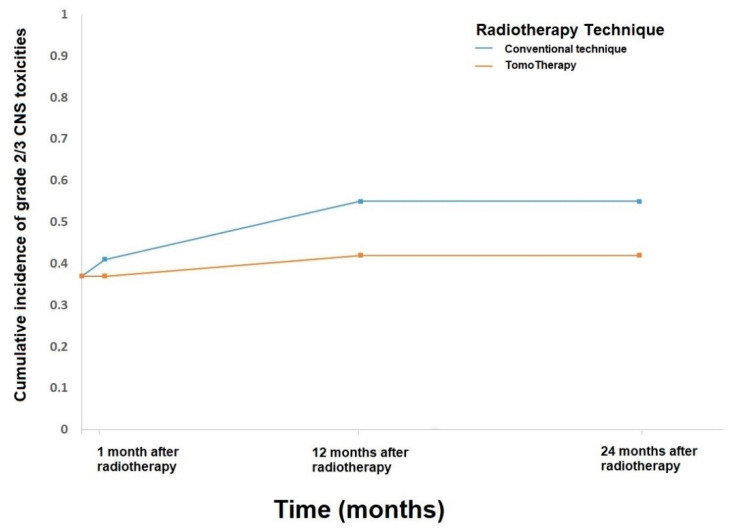
Cumulative incidence curve of grade 2/3 central nervous system (CNS) toxicities according to radiation technique.

Regarding the radiation technique, a trend towards less moderate (grades 2 and 3) upper gastrointestinal toxicities (*p* = 0.07) was detected with HT (n = 12/19; 63%) compared to conventional radiotherapy (n = 18/27; 67%). In terms of subacute toxicities, we detected a significantly lower CNS toxicity (*p* = 0.05) in the HT-group (n = 10/18; 56%) compared to CRT-group (n = 24/27; 89%). However, there were two patients with mild diarrhea (grade 1–2) 30 days following HT (compared to one patient in CRT-group, *p* = 0.1), which persisted up to 1-year follow-up (five patients in HT-group vs. one patient in CRT-group, *p* = 0.07). After 1 year of radiotherapy, the CNS toxicities risk was significantly lower following HT (11/17 patients in HT-group vs. 20/26 patients in CRT-group, *p* = 0.02). In a subgroup analysis, the rate of grade 2–3 toxicities was 29% (n = 5) in the HT-group vs. 58% (n = 15) in CRT-group. Six patients treated with CRT suffered from myelopathy (fiv grade 1 and one grade 2) versus no HT-group patients. At 2-year follow-up, we detected significantly higher moderate (grade 2/3) CNS toxicities in the CRT-group (12/25 patients) versus HT-group (1/10 patient, *p* = 0.003; Figure 2). This CNS toxicity remained significant at 3-year (1/9 patient vs. 11/23 patients, *p* = 0.02) despite the small sample size. All other AEs (Table 2) were not significantly different regarding the radiotherapy technique. 

Regarding the applied radiation dose, there was lower rate of grade 3–4 hematologic toxicity during radiotherapy following low-dose CSI (28% vs. 52%, *p* = 0.02) and a trend towards lower grade 2 skin toxicity (0% vs. 21%, *p* = 0.1) compared to high-dose CSI group. After one year, the rate of grade 2 lower GIT toxicity was also lower in the low-dose CSI group (0% vs. 4%, *p* = 0.05). All other AEs were not significantly different regarding the CSI dose.

In terms of second neoplasms, five patients (11%) experienced therapy-induced neoplastic lesions (four in the CRT-group vs. one patient in the HT-group). One patient developed myelodysplastic syndrome 6 years after treatment. Another patient experienced sphenoid wing meningioma grade 1 after 11 years. While, two patients developed brainstem glioblastoma 3 and 17 years after therapy. One patient experienced a malignant peripheral nerve sheath tumor at the level of the lumbar spine.

## 4. Discussion 

As medulloblastoma management evolved over the past decades, treatment regimens now clearly have curative intent, which shows the importance of acute and late treatment-related side effects and impact on patients’ quality of life. Many factors affect the prognosis of the treatment outcome, such as grade of tumor resection, tumor histology, biology, chemotherapy, and radiotherapy regimens [14,34,35,36]. Our analysis assessed CSI’s clinical outcome in medulloblastoma patients via HT compared to 3DCRT. Two critical findings emerged from this analysis: we detected acceptable survival rates regardless of the radiation technique and a lower rate of (upper gastrointestinal and central nervous system) toxicities following HT compared to 3DCRT.

We detected acceptable survival rates with 5-year OS and EFS of 95% and 93%, respectively. Several researchers have reported similar survival rates, with OS ranging between 86% and 100% and EFS ranging from 78% to 81% [5,13,37,38]. On the other hand, lower survival rates have also been reported, which could be explained by several reasons, like different RT doses or chemotherapy regimens, and small cohort sizes [36].

We could not detect any significant differences in EFS regarding the impact of initial metastasis at medulloblastoma diagnosis. In accordance with our data, Rieken et al. [36] did not observe the inferior outcome of patients with metastatic medulloblastoma. In contrast, von Bueren et al. report 5-year EFS rates of 62% in patients with metastatic medulloblastoma treated according to the HIT 2000 protocol [39].

Our data does not show any significant impact on 5-year EFS and 5-year OS regarding total gross resection of the tumor. The importance of total gross resection is still unclear and controversially discussed. A Spanish unicentric study found a correlation between total tumor resection and survival of patients [40]. Rieken et al. also state that, amongst others, complete tumor resection is associated with improved OS. To some part, these findings confirm the generally reported better survival rates of standard-risk MB patients in literature [5,14,15,38]. On the other hand, data from a German study [41] and Padovani et al. [42] support our findings, detecting no significant gross resection impact in adult and pediatric MB patients.

In total, 13% of our patients developed a recurrence during the follow-up period, with a median TTP of 22 months. Other studies show similar results regarding both incidence of relapse and TTP [5,15,38,43]. Our findings also show no significant difference between radiation techniques regarding relapse prevalence (16% vs. 11%, *p* = 1). 

HT performed better regarding grades 2 and 3 upper gastrointestinal toxicities than the conventional technique (63% vs. 67%, *p* = 0.07). This may be because the OAR sparing is better with HT [27,44]. Hong et al. support this with their study, showing that the volume of small bowel and esophagus receiving more than 10 Gy is significantly lower with HT [45]. 

Both radiation techniques are accompanied by long-term toxicities, being skin, CNS, and especially sensorineural hearing loss as the most common. Especially subacute CNS toxicities are significantly lower in the HT group (56% vs. 89%, *p* = 0.05). The first year after radiotherapy (65% vs. 77%, *p* = 0.02) CNS toxicities in general and after 2 years (10% vs. 48%, *p* = 0.003) grade 2–3 CNS toxicities are significantly higher in the CRT-group. Scobioala et al. [16] report superior hearing outcome in mid-high frequency with HT versus conventional radiotherapy. The reasons for lower CNS toxicities are, most likely, better outlining of the PTV due to daily pretreatment MVCT and the ability to irradiate the complex-shaped neuroaxis continuously, without the need for matching fields [13,18,20,27,29]. This ultimately results in higher dose conformity and homogeneity in the planning target volume [44]. As the acute toxicity rate may depend on the concomitant chemotherapy, we did not observe significant difference between the two groups regarding the applied concurrent chemotherapy regimen [46]. 

As a drawback of HT there are several reports that OAR volumes receiving low doses are higher in HT compared to the conventional technique [21,25,31]. As the ring gantry rotates around the patient, structures like the stomach receive higher amounts of low dose radiation. This explains our data, showing a trend towards higher rates of grade 1–2 diarrhea 1 month after HT (11% vs. 4%, *p* = 0.1), which persisted during the first year of follow-up examinations (29% vs. 4%, *p* = 0.07). 

Some authors raise concerns about higher integral doses in HT [27,47,48], leading to increased secondary neoplasms for patients receiving CSI via HT. Due to the short follow-up period of patients in the HT-group in our study, we did not compare both groups regarding the rate of secondary neoplasms. A few colleagues report that the integral dose is not increased or is even decreased by using HT compared to conventional radiotherapy [29,31,49]. Despite all these favorable results, the true importance of integral doses in secondary neoplasms is yet to be determined, as the mechanisms of radiocarcinogenesis are still not fully understood [50]. With an extended follow-up of more than 10 years, more studies are needed to make more accurate statements about the incidence of radiation-induced secondary tumors. 

Nonetheless, a significant point of critique of helical Tomotherapy is the prolonged beam-on time, which is described by several authors, resulting in longer treatment times, including the need for longer anesthesia or sedation [27,29,51,52]. 

As more and more patients survive medulloblastoma treatment, we must strive for novel treatment techniques to minimize AEs. More and more promising results were reported regarding proton beam therapy in the last years, resulting in lower toxicities for organs at risk [48,53,54]. Kahalley et al. report in a recent study better cognitive outcomes for patients treated with proton radiotherapy compared to patients treated with photons. The proton radiotherapy shows better outcomes in global IQ, perceptual reasoning, and working memory compared with the photon radiotherapy group [55]. This shows that we must consider proton beam therapy as a feasible alternative for treating young medulloblastoma patients. Just recently, in a pooled analysis of two French multicentric studies, intelligence could also be preserved in pediatric patients who received hyperfractionated photon radiation therapy and smaller boost volume than patients receiving standard regimens [12]. In the NOA-07 trial, combined radiochemotherapy followed by maintenance chemotherapy (cisplatin, lomustine, and vincristine) was accompanied by verbal working memory deterioration, while other neurocognitive domains were not impaired [56].

CSI dose reduction from 36 to 23.4 Gy did not decrease the irradiation’s effectiveness with a significant reduction in toxicity [57,58,59]. Therefore, in EORTC 1634-BTG (NOA-23) study, personalized risk-adapted therapy in adult medulloblastoma patients (>18 years old) with CSI with 23.4 Gy is currently under investigation [60]. Recently, an analysis of Children’s Oncology Group (COG) ACNS0331 [11] demonstrated that involved-field RT is noninferior to posterior fossa RT in all patients with average-risk medulloblastoma. In subgroup analysis, significant differences in outcome by molecular type were observed. In most of the children with medulloblastoma, CSI dose could be reduced to 18 Gy without loss of clinical benefits [11].

This study bears the limitation of being relatively small and retrospective, though the findings are clinically relevant to medulloblastoma patients. Furthermore, many clinical, molecular, and genetic characteristics were not available to assess its influence on treatment outcomes. Being a unicentric study limits our findings’ statistical power, albeit the present cohort’s size surpasses most single-center studies. Nevertheless, further prospective studies with larger patient sample and prolonged follow-up duration need to be done to fully comprehend HT’s benefits and disadvantages. More clinical and biological data are needed to identify patients who may require RT dose escalation as part of a biologically guided adaptive RT strategy.

## 5. Conclusions 

CSI via Tomotherapy is an efficacious treatment modality for patients with medulloblastoma. The rate of acute upper gastrointestinal and central nervous system toxicities might be reduced using HT.

## Figures and Tables

**Table 1:** Patient and treatment characteristics.

Characteristic	Nr. (% or Range)	Radiation Technique		
		Tomotherapy	Conventional	*p*-Value
Patients	46	19 (41%)	27 (59%)	
Med. age	7 (1–56)	7 (1–28)	8 (3–56)	0.37
Age group				0.2
≤ 4 years	12 (26%)	5 (26%)	7 (26%)	
>4–18 years	29 (23%)	10 (53%)	19 (70%)	
>18 years	5 (11%)	4 (21%)	1 (4%)	
Gender	M: 60 (65%) F: 16 (35%)	6 F (32%): 13 M (68%)	10 F (37%): 17 M (63%)	0.8
Metastasis				1
No	39 (85%)	16 (84%)	23 (85%)	
Yes	7 (15%)	3 (16%)	4 (15%)	
Histology				0.4
Classic	26 (63%)	15 (79%)	17 (63%)	
Desmoplastic	14 (34%)	4 (21%)	9 (33%)	
Anaplastic	1 (3%)	0 (0%)	1 (4%)	
Total resection				
Yes	27 (59%)	12 (63%)	15 (56%)	0.8
No	19 (41%)	7 (37%)	12 (44%)	
*Treatment protocol*				
HIT 2000	31 (67%)	7 (39%)	24 (89%)	0.001
Other	15 (33%)	12 (61%)	3 (11%)	
Radiation parameters				
Med. CSI dose, Gy	35 (23.4–40)	35 (23.4–40)	36 (23.4–40)	0.14
CSI with ≤24 Gy	14 (33%)	9 (47%)	5 (21%)	0.10
CSI ≥35 Gy	29 (67%)	10 (53%)	19 (79%)	
Med. fraction dose, Gy	1.8 (1–2)	1.8 (1–2)	1.7 (1–2)	0.7
Med. boost dose, Gy	55 (44–68)	55 (44–68)	56 (52–68)	0.5
Med. RT duration, days	53 (28–62)	59 (28–62)	47 (28–61)	0.5
Med. follow up, months	99 (5–249)	29 (5–92)	116 (25–249)	<0.001
Relapse pattern				1
**Yes**	6 (13%)	3 (16%)	3 (11%)	
Cerebral only	3/6	1/3	2/3	
Cerebral + spinal	3/6	2/3	1/3	
**No**	40 (87%)	16 (84%)	24 (89%)	

M, males; F, females; RT, radiotherapy; PTV, planning target volume.

**Table 2:** Acute and late radiation reactions of medulloblastoma craniospinal irradiation and the difference (*p*-value) according to conventional and Tomotherapy radiation techniques.

Toxicities Prior to Radiotherapy	N (%)	Grade 1	Grade 2	Grade 3	Grade 4	*p*-Value
Oral mucositis	3 (6%)	1 (2)	2 (4)	0	0	0.46
Conjunctivitis	1 (2)	1 (2)	0	0	0	1
CNS symptoms	31 (67)	15 (33)	11 (24)	5 (11)	0	0.75
Pharynx, esophagus	2 (4)	1 (2)	1 (1)	0	0	0.35
Larynx	1 (2)	1 (2)	0	0	0	0.4
Upper GIT	4 (8)	2 (4)	2 (4)	0	0	0.58
Lower GIT	4 (8)	2 (4)	2 (4)	0	0	0.05
Pneumonitis	2 (4)	1 (4)	0	1 (2)	0	0.48
Genitourinary	1 (2)	1 (2)	0	0	0	0.41
Heart	2 (4)	2 (4)	0	1 (2)	0	0.16
Hematological toxicity	16 (35)	2 (4)	8 (18)	5 (11)	1 (2)	0.17
**Acute toxicities**	**N (%)**	**Grade 1**	**Grade 2**	**Grade 3**	**Grade 4**	
Skin	43 (94)	33 (72)	9 (20)	1 (2)	0	0.38
Oral mucositis	13 (28%)	7 (15%)	6 (13%)	0	0	0.39
Conjunctivitis	2 (4)	2 (4)	0	0	0	0.5
CNS RT-related symptoms	31 (67)	14 (30)	14 (30)	3 (7)	0	0.42
Sensoryneural hearing loss	10 (21)	6 (13)	3 (6)	1 (2)	0	0.78
Salivary gland	5 (11)	5 (11)	0	0	0	0.14
Pharynx, esophagus	7 (15)	6 (13)	1 (2)	0	0	0.27
Larynx	1 (2)	1 (2)	0	0	0	1
Upper GIT	32 (69)	2 (4)	25 (54)	5 (11)	0	0.07
Lower GIT	8 (17)	7 (15)	1 (2)	0	0	0.16
Pneumonitis	3 (6)	2 (4)	0	1 (2)	0	0.16
Genitourinary	1 (2)	1 (2)	0	0	0	0.41
Heart	1 (2)	0	0	1 (2)	0	1
Hematological toxicity	33 (72)	5 (11)	8 (17)	10 (22)	10 (22)	0.1
**Subacute toxicities**						
Skin	42 (91)	36 (78)	6 (13)	0	0	0.3
Oral mucositis	6 (13%)	5 (11%)	1 (2%)	0	0	0.42
Conjunctivitis	1 (2)	0	1 (2)	0	0	1
CNS	34 (74)	16 (35)	13 (28)	5 (11)	0	0.05
Sensoryneural hearing loss	12 (26)	8 (17)	3 (7)	1 (2)	0	0.59
Salivary gland	2 (4)	2 (4)	0	0	0	1
Pharynx, esophagus	2 (4)	2 (4)	0	0	0	0.51
Larynx	1 (2)	1 (2)	0	0	0	1
Upper GIT	29 (63)	3 (6)	22 (48)	4 (9)	0	0.48
Lower GIT	3 (6)	2 (4)	1 (2)	0	0	0.15
Pneumonitis	1 (2)	0	0	1 (2)	0	1
Genitourinary	0	0	0	0	0	-
Heart	0	0	0	0	0	-
Hematological toxicity	24 (52)	5 (11)	7 (15)	10 (22)	2 (4)	0.14
**Chronic toxicities (at 12 months)**						
Skin	36 (78)	35 (76)	1 (2)	0	0	0.21
Oral mucositis	4 (8%)	2 (4%)	2 (4%)	0	0	0.9
Conjunctivitis	2 (4)	2 (4)	0	0	0	0.15
CNS	31 (67)	11 (24)	14 (30)	6 (13)	0	0.02
Sensoryneural hearing loss	23 (50)	5 (11)	17 (37)	1 (2)	0	0.19
Salivary gland	2 (4)	1 (2)	1 (2)	0	0	0.35
Pharynx, esophagus	4 (8)	1 (2)	2 (4)	1 (2)	0	0.31
Larynx	2 (4)	1 (2)	1 (2)	0	0	0.33
Upper GIT	32 (50)	0	21 (46)	2 (4)	0	0.2
Lower GIT	6 (13)	5 (11)	1 (2)	0	0	0.07
Pneumonitis	2 (4)	1 (2)	0	1 (2)	0	0.33
Genitourinary	1 (2)	1 (2)	0	0	0	1
Spinal cord	6 (13)	5 (11)	1 (2)	0	0	0.09
Heart	0	0	0	0	0	-
Hematological toxicity	25 (61)	6 (13)	2 (4)	8 (17)	14 (30)	0.18

RT: radiotherapy, GIT: gastrointestinal tract, CNS: central nervous system.

## Data Availability

The data presented in this study are available on request from the corresponding author. The data are not publicly available due to privacy.
